# Point-of-care ultrasound in the diagnosis of pulmonary embolism

**DOI:** 10.1186/s13089-015-0025-5

**Published:** 2015-05-27

**Authors:** Alessandro Squizzato, Luca Galli, Victor E. A. Gerdes

**Affiliations:** Research Center on Thromboembolic Disorders and Antithrombotic Therapies, Department of Clinical and Experimental Medicine, University of Insubria, Varese, Italy; Department of Medicine, Slotervaart Hospital, Amsterdam, The Netherlands; Department of Vascular Medicine, Academic Medical Centre, Amsterdam, The Netherlands; U.O. Medicina I, Ospedale di Circolo, Viale Borri 57, 21100 Varese, Italy

**Keywords:** Pulmonary embolism diagnosis, Venous thromboembolism, Point-of-care ultrasound, Lung ultrasound, Echocardiography, Lower limb compressive venous ultrasonography

## Abstract

The best diagnostic strategy to confirm or exclude pulmonary embolism (PE) suspicion needs an appropriate combination of clinical assessment, plasma D-dimer measurement, and computed tomographic pulmonary angiography (CTPA). CTPA should be used with caution in some patient groups, such as patients with known allergy to contrast media, those with severe renal insufficiency, and pregnant women, and could be not immediately available in case of unstable patients. In the emergency setting, alternative diagnostic strategies should be implemented to overcome CTPA limitations. Ultrasonography is certainly a valuable alternative diagnostic tool. In addition to echocardiography and lower limb compressive venous ultrasonography, lung ultrasound (US) may play an important role in selected patients’ subgroups. Recent data on the diagnostic performance of a triple point-of-care US (lung, heart, and leg vein US) are discussed in the present paper, and pros and cons of triple point-of-care US are compared with those of standard diagnostic approaches.

## Introduction

Venous thromboembolism (VTE) is a major health problem, with an overall annual incidence between 100 and 200 per 100,000 inhabitants [1]. Acute pulmonary embolism (PE) is the most serious clinical presentation of VTE and may be life-threatening or lead to chronic pulmonary hypertension if not early diagnosed and treated [2]. Signs and symptoms of PE are non-specific, and several cardiopulmonary diseases should be taken into account in the differential diagnosis: no laboratory or imaging test has a sufficient accuracy to be used as a single test for this complex diagnostic workup [3]. The European Society of Cardiology (ESC) suggests, in the last edition of guidelines on the diagnosis and management of acute pulmonary embolism, that the best diagnostic strategy to confirm or exclude PE suspicion needs an appropriate combination of clinical assessment, plasma D-dimer measurement, and computed tomographic pulmonary angiography (CTPA) [2].

## Review

CTPA has brought a great improvement in the diagnostic approach to patients with suspected PE, allowing an adequate visualization of the pulmonary arteries and their thromboembolic obstruction up to at least the segmental level, with a high specificity and sensitivity [4]. On the other hand, the predictive value of CTPA is influenced by clinical probability, as shown in PIOPED II, with a much higher negative predictive value of a negative CT in patients with a low or intermediate pre-test likelihood in comparison to patient with a high clinical probability assessed by the Wells rule [5]. This suggests that a negative CTPA can be sufficient to exclude PE in patients with a non-high clinical probability of PE, but whether patients with negative CTPA and high clinical probability should be further investigate is controversial [2]. The increased and widespread use of CTPA in patients with suspected PE in these years has led to an observed increase in the diagnosis of PE, without a corresponding decline in mortality rate [6]. This could be likely explained by an increase in the diagnosis of small, subsegmental, and non-fatal emboli and may raise some concern about an appropriate selection of patients suspected for PE who underwent CTPA [7]. On the other hand, even the increasing use of CTPA has not affected mortality, it may have allowed earlier diagnosis and treatment, with shorter hospital stay.

Nevertheless, CTPA should be used with caution in some patient groups, such as patients with known allergy to contrast media, those with severe renal insufficiency, and pregnant women, and could be not immediately available in case of unstable patients. Hence, in the emergency setting, alternative diagnostic strategies should be implemented to overcome these limitations [2]. Ultrasonography is certainly a valuable alternative diagnostic tool. Indeed, the ESC guidelines suggest that echocardiography and lower limb compressive venous ultrasonography (CUS), but not lung ultrasound (US), may play an important role in selected patients’ subgroups.

Echocardiography has been extensively investigated in PE patients [2]. It is a valuable prognostic tool for stratifying PE patients with or without right ventricular dysfunction, in particular if combined with clinical assessment, i.e., the Pulmonary Embolism Severity Index (PESI), and blood tests, i.e., troponin and natriuretic peptide. Besides, it is really useful in patients with shock or persistent hypotension with clinical signs of acute right heart failure, e.g., jugular vein dilatation. Indeed, echocardiography rarely may directly visualize emboli in right cavities and in the pulmonary artery. Therefore, echocardiography is usually recommended in those cases of suspected high-risk PE in which the patient’s conditions are so critical that only bedside diagnostic tests are allowed [2, 8]. In these cases, if CTPA is not immediately available or feasible, finding clear and undeniable signs of right ventricular dysfunction and pressure overload without other causes of acute right dysfunction, e.g., cardiac tamponade or right myocardial infarction, allows emergency primary reperfusion treatment. On the other hand, PE is virtually ruled out as the cause of hemodynamic instability in the absence of those signs, and further causes of shock can be searched with the help of transthoracic echocardiography [2]. However, in patients without shock or hypotension, a negative finding on echocardiography cannot rule out PE, due to the reported low negative predictive value of the test [9].

Lower limb CUS is certainly an optimal diagnostic tool for both unstable and stable PE patients, since that a proximal positive result of the test has a high positive predictive value for PE [10]. CUS may identify a deep vein thrombosis (DVT) in up to half of patients with PE [11], and due to its high positive predictive value, finding proximal DVT in a patient suspected of PE allows to start an anticoagulant treatment unless contraindicated, e.g., concomitant bleeding or very high risk of bleeding [2]. Like echocardiography, lower limb CUS is unable to exclude PE due to its low sensitivity and low negative predictive value [9]. Therefore, the diagnosis should be finally confirmed by an imaging test of the lung as soon as the unstable patient has been stabilized or CTPA is available for stable patients [2]. In addition, CUS is a valuable option in those stable patients suspected for not high-risk PE with relative or absolute contraindications for CTPA, such as in renal failure, allergy to contrast media, or pregnancy [12]. Also, in these patients’ subgroups, PE cannot be confirmed or excluded with a high probability, but anticoagulation treatment cannot be delayed.

Lung US role is not discussed in the ESC guidelines, even though several clinicians routinely use this diagnostic tool in patients presenting with dyspnoea and/or chest pain every day. Peripheral parenchymal consolidations can be visible on lung ultrasound when an embolic vascular occlusion occurs. These consolidations are due either to necrosis of lung parenchyma (infarction) or to atelectasis, related to breakdown of surfactant with extravasation of blood. Since the first description of ultrasound morphology of pulmonary infarction in the 1960s, several diagnostic accuracy studies have been published [13]. Mathis et al. showed that lung US could demonstrate subpleural pulmonary consolidations due to embolism in more than 75 % of patients having a PE [14]. A recent systematic review of accuracy test studies of lung US for the diagnosis of PE in patients with clinical suspicion of PE estimated a sensitivity of 87.0 % and a specificity of 81.8 % when this technique was used as a single test [15]. Pooled sensitivity and specificity are similar to those found with single- and two-row detector CTPA but inferior to the current used multidetector CTPA [7]. However, lung US accuracy was better in low-quality studies than in the ones with high quality [15]. Based on these data, lung US cannot be considered as the first imaging test but a possible alternative to CTPA when the latter is contraindicated. Lung US has several advantages in comparison to other imaging tests, including the absence of biological risks for the patient and virtually no contraindications. Moreover, lung US can be safely used in case of both renal insufficiency and pregnancy. Lastly, as a bedside test, it can extremely useful in hemodynamically unstable patients.

However, some limitations exist and can reduce the accuracy of this test when used alone. Even though most of the pulmonary infarctions were detected in the lower lobes [14], only two thirds of the lung area is easily accessible and more central lesions can be missed. Moreover, when the embolic vascular occlusion causes solely the so-called “early infarction” or “pulmonary hemorrhage” and not a lung infarction, the related alterations may remain visible on ultrasound for only few hours after PE occurrence. To the best of our knowledge, no theoretical technical improvement may overcome this and other biological limits, such as the rapid ultrasound dissipation by air. In addition, lung US, as any other ultrasonographic test, is operator-dependent, and a specific and appropriate training is necessary. Finally, no published study specifically investigated lung US accuracy in PE patients’ subgroups, such as unstable patients and pregnant women.

Given that the accuracy of any diagnostic method is highly enhanced when used in conjunction with others, clinicians have advocated that a proper combination of US methods may theoretically improve the diagnostic accuracy, in particular for specific PE clinical presentation and patients’ subgroups. In the emergency department and intensive care setting, such a combined strategy has already improved clinical practice. The application of the BLUE protocol, where lung US has been used in conjunction with lower limb CUS in critically ill patients, may diagnose PE with 81 % sensitivity and 99 % specificity after the exclusion of other causes of severe respiratory failure [16]. Anyway, the BLUE protocol, looking only at indirect signs of PE, can be more useful in differentiating causes of dyspnoea in acute respiratory failure patients than in confirming or excluding PE diagnosis in patients with suspected PE. Indeed, the BLUE protocol has been tested in combination with echocardiography in a small study performed in an emergency department/inpatient medical service setting [17]. Ninety-six non-consecutive patients in whom a CTPA was ordered to rule out PE underwent a limited echocardiography, lung US, and lower limb CUS, before or within 3 h from CTPA execution. No PE was identified by CTPA in any of the 56 patients who was judged not to need a CTPA based on extended US examination results alone. On the other hand, PE was diagnosed by CTPA in 30 % of the 40 patients in whom the CTPA was judged useful. Basing on these findings, the authors conclude that extended ultrasonography approach may be useful in excluding the diagnosis of PE but not in making the diagnosis of PE.

Concomitantly, the quality improvement of ultrasound equipment, with increasingly compact sizes and lower costs, has made the idea of an “ultrasound stethoscope” more close to reality, allowing the growth and spread of point-of-care US (POC-US), performed and interpreted by the clinician at the bedside [18]. The POC-US strategy was recently tested by Nazerian and colleagues for PE diagnosis with positive results [19]. In this study, the diagnostic performance of a triple POC-US (lung, heart, and leg vein US) was investigated in 357 patients with clinical suspicion of PE with a Wells score >4 or a positive D-dimer value. The sensitivity and the specificity of this combined approach were 90 % and 86.2 %, respectively. Moreover, this combined triple POC-US approach identified an alternative diagnosis in almost one third of included patients and, in particular, in almost half of patients in whom PE was excluded [19].

Triple POC-US is highly promising. It can be performed at bedside providing real-time dynamic images that can be directly correlated with the patient’s clinical features, in particular in unstable PE patients. It can also be repeated in case of evolving patient’s condition, without clinically relevant biological risks for the patient. Furthermore, portable ultrasound systems are even more widespread, both in emergency and in critically ill patients departments and in internal medicine departments, making the use of this technique more feasible. Lastly, but not less relevant, this multiorgan ultrasonographic approach can often allow the achieving of an alternative diagnosis to PE. These features could make the POC ultrasound approach a more reliable alternative to CTPA for the PE diagnosis when CTPA is contraindicated or not available than the single-organ US approach.

Nevertheless, some issues should be discussed before implementing the POC-US protocol in the current clinical practice. First, each single US had lower accuracy in comparison with the triple US approach, with lung US as the best performer with a sensitivity of 61 % [19]. These data confirm limitations of a single-organ US approach in ruling out PE but suggest that the sensitivity of lung US is lower than that reported in previous studies [15]. Several reasons may explain this difference. In the emergency departments, the limited time available for US and the clinical status of patients make difficult to scan the whole chest, missing frequently the dorsobasal segments of the lung, where the majority of embolic pulmonary consolidations can be found. Second, there are no data on PE and DVT recurrence in the first 3 months after a negative diagnostic work-up, as previously done with CTPA [19, 20]. Third, as an operator-dependent technique, triple POC-US requires a specific and appropriate training. As underlined by Nazerian and colleagues, the use of the same methodology by less experienced physicians may lead to lower accuracy and safety. Fourth, patients should anyhow undergo an adequate clinical assessment to identify specific clinical presentation suspicious for PE. Indeed, an indiscriminate use of US could result in further unnecessary testing, unnecessary and potentially harmful interventions in the case of false positive findings, or deficient investigation of false negative findings [18]. Lastly, even though a potential application of this new approach could be exactly in patients with contraindications to CTPA, those patients were excluded from Nazerian and colleagues’ study.

## Conclusions

Given these issues, further and extensive clinical studies are needed. These studies should focus on specific patients’ subgroups, e.g., patients with known allergy to contrast media, with severe renal insufficiency, pregnant women, and unstable patients, or integrate triple POC-US with existing diagnostic algorithms. Indeed, Nazerian and colleagues are already testing the hypothesis that triple POC-US may be useful in the selection of patients with suspected PE who should undergo CTPA, improving the potential of the combination of Wells score and D-dimer test [21].

The new era of triple POC-US has just started.

## References

[1] Cohen AT, Agnelli G, Anderson FA, Arcelus JI, Bergqvist D, Brecht JG, Greer IA, Heit JA, Hutchinson JL, Kakkar AK, Mottier D, Oger E, Samama MM, Spannagl M, VTE Impact Assessment Group in Europe (VITAE) (2007). Venous thromboembolism (VTE) in Europe. The number of VTE events and associated morbidity and mortality. Thromb Haemost.

[2] Konstantinides SV, Torbicki A, Agnelli G, Danchin N, Fitzmaurice D, Galiè N, Gibbs JS, Huisman MV, Humbert M, Kucher N, Lang I, Lankeit M, Lekakis J, Maack C, Mayer E, Meneveau N, Perrier A, Pruszczyk P, Rasmussen LH, Schindler TH, Svitil P, Vonk Noordegraaf A, Zamorano JL, Zompatori M (2014). ESC Guidelines on the diagnosis and management of acute pulmonary embolism: the Task Force for the Diagnosis and Management of Acute Pulmonary Embolism of the European Society of Cardiology (ESC) Endorsed by the European Respiratory Society (ERS). Eur Heart J.

[3] Squizzato A, Luciani D, Rubboli A, Di Gennaro L, Landolfi R, De Luca C, Porro F, Moia M, Testa S, Imberti D, Bertolini G (2013). Differential diagnosis of pulmonary embolism in outpatients with non-specific cardiopulmonary symptoms. Intern Emerg Med.

[4] Ghaye B, Szapiro D, Mastora I, Delannoy V, Duhamel A, Remy J, Remy-Jardin M (2001). Peripheral pulmonary arteries: how far in the lung does multi-detector row spiral CT allow analysis?. Radiology.

[5] Stein PD, Fowler SE, Goodman LR, Gottschalk A, Hales CA, Hull RD, Leeper KV, Popovich J, Quinn DA, Sos TA, Sostman HD, Tapson VF, Wakefield TW, Weg JG, Woodard PK, PIOPED II Investigators (2006). Multidetector computed tomography for acute pulmonary embolism. N Engl J Med.

[6] Burge AJ, Freeman KD, Klapper PJ, Haramati LB (2008). Increased diagnosis of pulmonary embolism without a corresponding decline in mortality during the CT era. Clin Radiol.

[7] Wiener RS, Schwartz LM, Woloshin S (2013). When a test is too good: how CT pulmonary angiograms find pulmonary emboli that do not need to be found. BMJ.

[8] Kucher N, Luder CM, Dornhofer T, Windecker S, Meier B, Hess OM (2003). Novel management strategy for patients with suspected pulmonary embolism. Eur Heart J.

[9] Roy PM, Colombet I, Durieux P, Chatellier G, Sors H, Meyer G (2005). Systematic review and meta-analysis of strategies for the diagnosis of suspected pulmonary embolism. BMJ.

[10] Le Gal G, Righini M, Sanchez O, Roy PM, Baba-Ahmed M, Perrier A, Bounameaux H (2006). A positive compression ultrasonography of the lower limb veins is highly predictive of pulmonary embolism on computed tomography in suspected patients. Thromb Haemost.

[11] Kearon C, Ginsberg JS, Hirsh J (1998). The role of venous ultrasonography in the diagnosis of suspected deep venous thrombosis and pulmonary embolism. Ann Intern Med.

[12] Righini M, Le Gal G, Aujesky D, Roy PM, Sanchez O, Verschuren F, Kossovsky M, Bressollette L, Meyer G, Perrier A, Bounameaux H (2009). Complete venous ultrasound in outpatients with suspected pulmonary embolism. J Thromb Haemost.

[13] Miller LD, Joyner CR, Dudrick SJ, Eskin DJ (1967). Clinical use of ultrasound in the early diagnosis of pulmonary embolism. Ann Surg.

[14] Mathis G, Blank W, Reissig A, Lechleitner P, Reuss J, Schuler A, Beckh S (2005). Thoracic ultrasound for diagnosing pulmonary embolism: a prospective multicenter study of 352 patients. Chest.

[15] Squizzato A, Rancan E, Dentali F, Bonzini M, Guasti L, Steidl L, Mathis G, Ageno W (2013). Diagnostic accuracy of lung ultrasound for pulmonary embolism: a systematic review and meta-analysis. J Thromb Haemost.

[16] Lichtenstein DA, Meziere GA (2008). Relevance of lung ultrasound in the diagnosis of acute respiratory failure: the BLUE protocol. Chest.

[17] Koenig S, Chandra S, Alaverdian A, Dibello C, Mayo PH, Narasimhan M (2014). Ultrasound assessment of pulmonary embolism in patients receiving CT pulmonary angiography. Chest.

[18] Moore CL (2011). Copel. Point-of-care ultrasonography. N Engl J Med.

[19] Nazerian P, Vanni S, Volpicelli G, Gigli C, Zanobetti M, Bartolucci M, Ciavattone A, Lamorte A, Veltri A, Fabbri A, Grifoni S (2014). Accuracy of point-of-care multiorgan ultrasonography for the diagnosis of pulmonary embolism. Chest.

[20] Huisman MV, Klok FA (2009). Diagnostic management of clinically suspected acute pulmonary embolism. J Thromb Haemost.

[21] Nazerian P et al. Pulmonary embolism diagnosis: ultrasound Wells score versus traditional Wells score. www.clinicaltrials.gov. Accessed on 23 December 2014

